# XMU-MP-1, Inhibitor of STE20-like MST1/2 Kinases of the Hippo Signaling Pathway, Suppresses the Cell Cycle, Activates Apoptosis and Autophagy, and Induces Death of Hematopoietic Tumor Cells

**DOI:** 10.3390/ph18060874

**Published:** 2025-06-12

**Authors:** Alexander G. Stepchenko, Sofia G. Georgieva, Elizaveta V. Pankratova

**Affiliations:** 1 Engelhardt Institute of Molecular Biology, Russian Academy of Sciences, Vavilov Str. 32, 119991 Moscow, Russia; step@eimb.ru (A.G.S.);; 2 Center for Precision Genome Editing and Genetic Technologies for Biomedicine, Engelhardt Institute of Molecular Biology, Russian Academy of Sciences, Vavilov Str. 32, 119991 Moscow, Russia

**Keywords:** MST1/2 kinases inhibitor, hematopoietic tumors, programmed cell death, XMU-MP-1, cell cycle, cancer resistance, Hippo signaling pathways

## Abstract

**Background/Objectives**: Currently, there is limited knowledge on the molecular mechanisms of the “non-canonical” Hippo signaling pathway in hematopoietic tumor cells. We have shown that targeting the MST1/2 kinases, which are the key molecules in this signaling pathway, may be an effective approach to the treatment of hematologic tumors. **Methods**: The methods used in this study include cell growth assays, caspase assays, Western blot hybridizations, flow cytometry, and whole-transcriptome analyses. These methods allowed us to better understand the molecular pathways at play. **Results**: Our results showed that XMU-MP-1, an inhibitor of MST1/2 kinase, specifically reduces the viability of hematopoietic cancer cells but not breast cancer cells. It effectively inhibits the growth of the tumor B- and T-cell lines by blocking cell cycle progression, mainly during the G2/M phase, inducing apoptosis and autophagy. XMU-MP-1 treatment led to increased caspase 3/7 activity and increased levels of the cleaved PARP protein. Levels of the LC3-II protein were also shown to be increased, while the level of p62 decreased. These changes are associated with apoptosis and autophagy, respectively. RNA-seq analysis has demonstrated that XMU-MP-1 suppressed the expression of cell cycle regulators, such as E2F, and cell division cycle genes CDC6,7,20,25,45; cyclins A2,B1,B2, and cyclin-dependent kinases. At the same time, it increased the expression of genes involved in apoptosis, autophagy, and necroptosis. **Conclusions**: Combinations of growth assays, caspase assays, Western blotting, and RNA-seq have shown that the dramatic reduction in the number of hematopoietic tumor cells after treatment with XMU-MP-1 is due to both cytostatic and cytotoxic effects. The use of MST1/2 kinase inhibitors could be highly promising for complex therapy of hematological tumors.

## 1. Introduction

Leukemia and lymphoma are common hematologic malignancies in children, young people, and adults which pose a huge threat to the lives of these patients worldwide, despite the availability of efficient therapies. Finding ways to cure these diseases is of great importance in oncohematology.

In this study, we show that targeting MST1/2 kinases, the key molecules in the Hippo signaling pathway, may prove to be an effective way to treat hematologic tumors.

The Hippo signaling pathway regulates the development of organs and their size, tissue regeneration, and apoptosis. The central role of this pathway in maintaining tissue homeostasis has been demonstrated [1]. Disorders in this signaling pathway usually lead to tumor formation [2,3]. A detailed analysis of the Hippo signaling pathway is extremely hard to perform due to it including a multitude of components and its ability to respond to a vast array of extracellular and intracellular signals [4]. The key players in the Hippo signaling pathway include MST1/2 and LATS1/2 kinases and their targets, YAP/TAZ transcription coactivators [4,5,6]. LATS1/2 is phosphorylated by the MST1/2–SAV1 complex [7]. Being thereby activated, in turn, it phosphorylates and inactivates YAP and TAZ [8,9,10]. If YAP/TAZ are not phosphorylated, they are translocated to the nucleus and act via TEAD 1-4 and other transcription factors that they interact with [11,12]. Disruption of this signaling pathway is observed in many human tumors. A recent systematic analysis of tumor samples revealed dysregulation of the Hippo signaling pathway components in many types of human cancers, including glioma, colorectal cancer, endometrial cancer, and hepatocellular carcinoma [2,10,13]. YAP/TAZ hyperactivation stimulates cancer cell proliferation [14]. Cells stop responding to the negative regulators of proliferation, overcome internal death mechanisms, and acquire resistance to chemotherapeutic drugs or molecular targeted therapy, which contributes to cancer recurrence [15,16]. However, the way that the Hippo signaling pathway functions depends on the cell type. For example, in hematopoietic cells, the pathway works in an atypical way, or an alternative variant is used. Unlike solid tumors, knocking down MST1 or increasing YAP1 expression in hematopoietic tumors inhibits growth and leads to apoptosis [17]. It has recently been shown that treatment with YAP activators suppresses cell growth in leukemia cell lines, with apoptotic induction involving an increase in cleaved caspase-3. Furthermore, this treatment downregulates the expression of NOTCH1 and cleaved NOTCH1, as well as MYC [18]. In leukemia, lymphoma, and multiple myeloma, low YAP1 levels block apoptosis, while YAP1 activation causes the death of hematologic cancer cells. Genetic inactivation of MST1 keeps YAP1 unphosphorylated and triggers apoptosis in vitro and in vivo [17]. These data demonstrate that MST1 can be a promising therapeutic target, and can provide a rationale for the development and clinical evaluation of novel MST1 inhibitors. The CN103429582A patent describes a drug called XMU-MP-1. This compound is a reversible, potent, and selective inhibitor of Mammalian sterile 20-like kinases 1/2 (MST1/2), the key molecules in the Hippo signaling pathway. XMU-MP-1 blocks the activity of MST1 and MST2 kinases, which reduces the phosphorylation of endogenous LATS1/2 and YAP1 in human liver carcinoma (HepG2) cells when used in the concentrations of 0.1 to 10 μM in a dose-dependent manner. This leads to the activation of YAP1 and TAZ and their nuclear translocation. Similar results have been obtained for other tumor cell lines. XMU-MP-1 was originally designed and used as a “small molecule” to analyze the Hippo signaling pathway [19,20] and as a drug promoting liver regeneration, as well as for the prevention and treatment of hematopoietic system diseases caused by oxidative stress and ionizing radiation. XMU-MP-1 treatment restores hematopoietic stem cell and progenitor cell functions under the oxidative stress induced by ionizing radiation and increases the survival rate of mice which had received lethal radiation doses [20]. Earlier, we demonstrated for the first time the anticancer activity of XMU-MP-1 against a tumor B-cell line [21].

In this study, we showed that XMU-MP-1 effectively inhibits the growth of B- and T-cell hematologic tumor cells. XMU-MP-1 blocks the cell cycle in the G2/M phase, inducing cell death, apoptosis, and autophagy. It also enhances the effects of doxorubicin. Whole-transcriptome analysis revealed the key regulatory genes involved in these processes, the expression of which is changed in response to the XMU-MP-1 treatment.

## 2. Results

### 2.1. Hippo Pathway Inhibitor XMU-MP-1 Suppresses the Growth of B and T Tumor Cells

**Figure 1:**
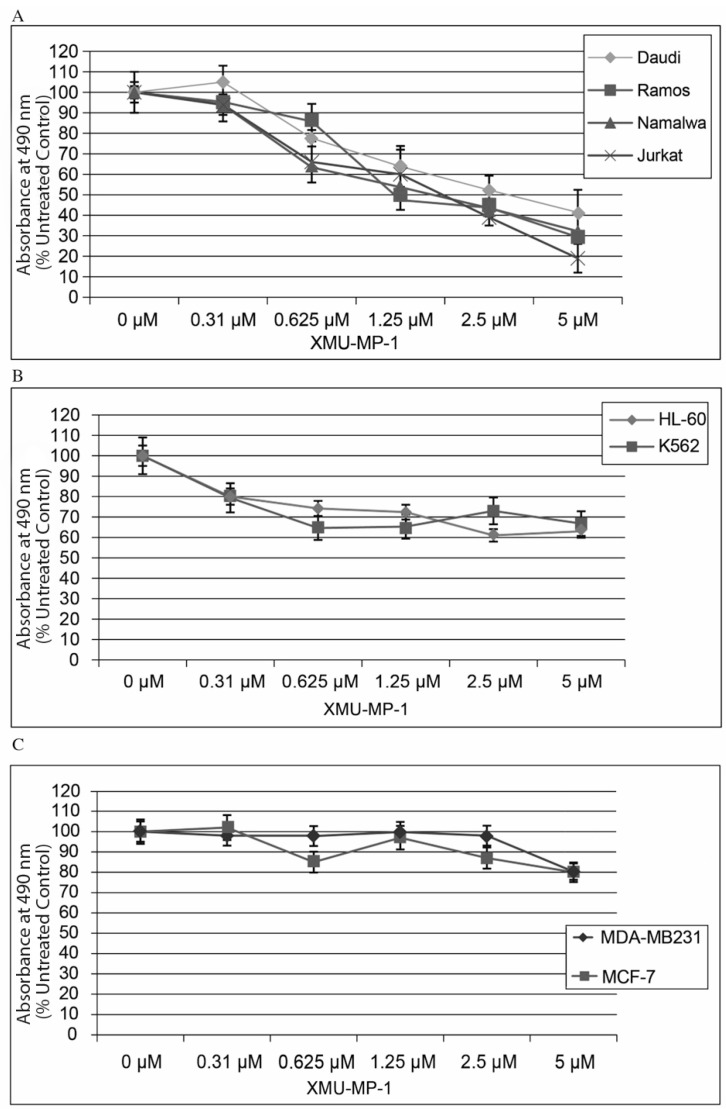
XMU-MP-1 effect on cell growth rate. (**A**). T and B cells. (**B**). Myeloid leukemia cells. (**C**). Breast cancer cells. Cells were grown in the complete DMEM medium in 96-well plates in four replicates for each line and each time point. The plots show mean ± S.E.M. of four independent experiments.

Using the CellTiter Aqueous One Solution kit, we have shown that treatment of hematopoietic cell lines with the MST1/2 inhibitor XMU-MP-1 results in a concentration-dependent decrease in the proportion of viable cells (Figure 1). This XMU-MP-1 effect was particularly strong in the B- and T-tumor cell lines (Figure 1A). EC50 was achieved in the Namalwa, Raji, Ramos, Jurkat, and Daudi cell lines in 72 h at XMU-MP-1 concentrations ranging from 1.21 to 2.7 µM (Figure 1A, Supplementary Table S1). At the same time, HL-60 and K562 cell lines showed higher resistance to XMU-MP-1 (Figure 1B). The breast cancer cell lines MDA-MB231 (triple-negative) and MCF-7 (triple-positive) were resistant to XMU-MP-1 in the discussed concentration range (Figure 1C).

We performed an analysis of MST1 expression in the cell lines used in this work, which demonstrated high levels of MST1 expression in the Namalwa, Daudi, and Ramos B-cell lymphoblastomas and Jurkat T-cell lymphoblastoma (Supplementary Figure S1).

The CellTiter Aqueous One Solution kit (MTT/MTS analysis) is a common method used to study cell proliferation and death. However, the results obtained using this kit may reflect not only the changes in the number of cells, but also the changes in the metabolic activity of the cell population. To confirm that the observed effects were associated with a decrease in cell population numbers, we performed direct counting of Namalva cells treated with XMU-MP-1 and the control cells at 24, 48, and 72 h post-treatment. We found that XMU-MP-1 suppressed the growth of the cell population (Supplementary Figure S2).

### 2.2. XMU-MP-1 Induces Apoptosis in Hematopoietic Tumor Cells

**Figure 2:**
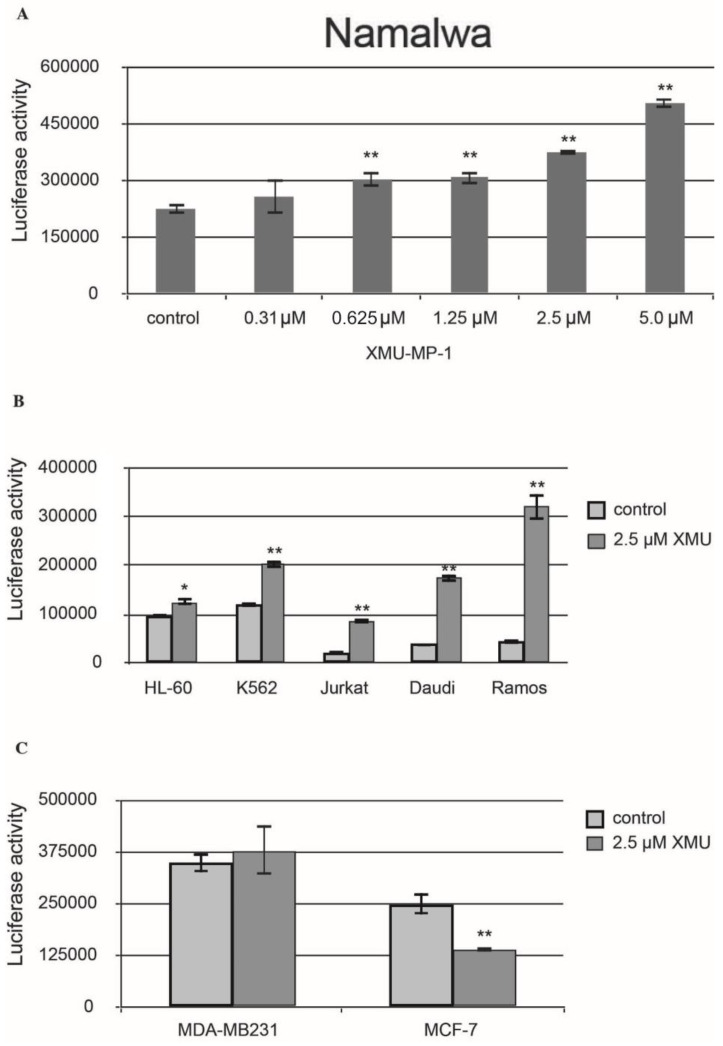
XMU-MP-1 effect on apoptosis in human cancer cell lines. (**A**) The concentration-dependent effect of XMU-MP-1 on the caspase 3/7 activity in the Namalwa cells. (**B**) Activity of caspase 3/7 in hematopoietic tumor cell lines. (**A**,**B**) Cells were inoculated into the 96-well plates at 30,000 cells/well in the final volume of 100 µL. (**C**) Caspase 3/7 levels in the breast cancer cell lines. Cells were inoculated into the 96-well plates at 15,000 cells/well in the final volume of 100 µL. Caspase 3/7 activity is plotted along the Y-axis. Apoptosis levels were assessed based on the caspase 3/7 activity estimated using the bioluminescent Caspase 3/7-Glo assay. The plots show mean ±S.E.M. for three independent experiments. *t*-tests were used to compare the means, and the asterisks indicate *p*-values relative to the control non-treated cells (* *p* < 0.05, ** *p* < 0.01). The measurements were made and images were obtained 24 h after the XMU-MP-1 treatment.

We then demonstrated that XMU-MP-1 causes apoptosis in Namalwa cells. The Caspase-Glo 3/7 assay protocol was utilized in this experiment. The analysis demonstrated a significant concentration-dependent increase in caspase 3/7 activity in the XMU-MP-1–treated Namalwa cell line (Figure 2A).

XMU-MP-1 also causes apoptosis in the Daudi, Ramos, Jurkat, K562, HL-60, and MP-1 cell lines. However, the XMU-MP-1-induced increase in caspase 3/7 activity may significantly differ between these tumor cell lines (Figure 2B). It is important to mention that XMU-MP-1 does not induce caspase 3/7 activation and apoptosis in the breast cancer MDA-MB231 and MCF-7 cell lines. The suppression of caspase 3/7 activity can even be observed in the MCF-7 cells, which indicates the suppression of apoptosis as a result of XMU-MP-1 application (Figure 2C).

To summarize, the results of our experiments have demonstrated that the response of cells to XMU-MP-1 is largely cell-specific. Antiproliferative and proapoptotic responses were predominantly found in the B- and T-cell lines.

### 2.3. RNA-Seq

Herein, to better understand the molecular mechanisms of the effects exerted by the Hippo signaling pathway inhibitor XMU-MP-1 on the B-cell lymphoma, we carried out a whole-transcriptome analysis and identified genes whose expression changed upon the Namalwa cell line treatment with XMU-MP-1. We also determined the biological functions of these genes and the underlying mechanisms of XMU-MP-1’s effect on the Namalwa cells. To do this, we prepared cDNA libraries from the control Namalwa cells and the Namalwa cells which were treated with XMU-MP-1 in the concentrations of 0.3 µM and 2.5 µM for 72 h.

Human mRNA profiles of the control Namalwa cells and the Namalwa cells treated with 0.3 or 2.5 μM of XMU-MP-1 were generated using new-generation sequencing on the Illumina NovaSeq platform. RNA-seq was used to identify differentially expressed genes (DEGs) in the cells treated with XMU-MP-1 (GEO database: GSE80287). Changes in gene transcription levels were considered significant based on a fold change ≥2.0 and a Paji ≤ 0.01.

DEGs, like the 0.3 µM and 2.5 µM XMU-MP-1 treatments, had a similar response in terms of the direction (either upward or downward change); however, the response levels were different. We also observed that the changes in gene activity were more pronounced at higher drug concentrations, with the effect levels differing by several times in some cases (Supplementary Tables S2–S5). This shows that the action of XMU-MP-1 on gene expression is concentration-dependent.

### 2.4. Functional Enrichment Analysis of DEGs

To identify biological processes and to determine more relevant groups of DEGs regulated by XMU-MP-1, we performed functional enrichment analysis by DAVID utilizing the GO (Gene Ontology) and KEGG pathways databases. Three gene ontology domains (biological process, cellular component, and molecular function) were analyzed for DEGs.

The functional enrichment analysis demonstrated XMU-MP-1 to be a negative regulator of cell cycle and a positive regulator of programmed cell death in hematopoietic tumor cells.

**Figure 3:**
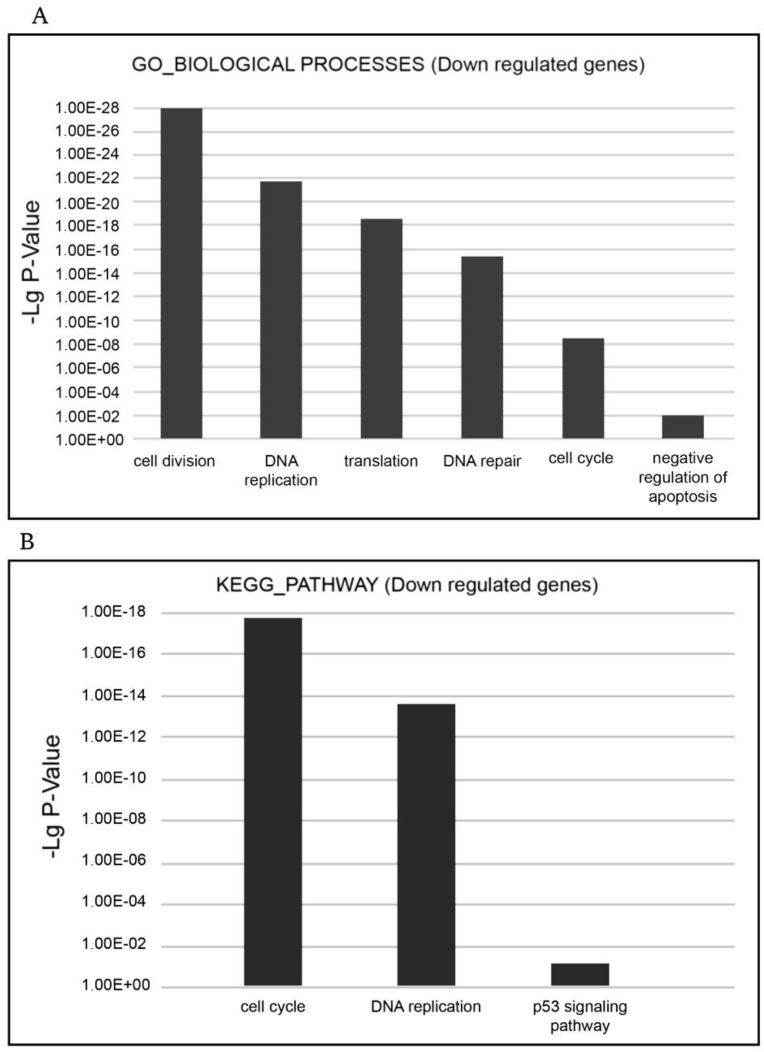
Functional enrichment analysis of downregulated differentially expressed genes (DEGs) in the XMU-MP-1-treated Namalwa cell line. (**A**) The most relevant BIOLOGICAL PROCESSES (Gene Ontology (GO) GOTERM BP FAT) and (**B**) the most relevant KEGG pathways.

The most relevant downregulated processes from the GO_BIOLOGICAL PROCESSES databases were ‘cell cycle’, ‘cell division’, ‘mitosis’, ‘DNA replication’, ‘DNA repair’, ‘DNA damage’, ‘mRNA splicing’, and ‘stress response’. The most relevant downregulated KEGG pathways were ‘cell cycle’, ‘DNA replication’, and ‘p53 signaling pathway’, which implied that negative cell cycle regulation is due to the XMU-MP-1 function in the Namalwa cells (Figure 3A,B).

The ‘cell cycle’ term encompasses a significant group of downregulated DEGs that participate in cell cycle regulation (Supplementary Table S2). This group includes the E2F family of transcription factors (E2F1, E2F2, E2F7, and E2F8), the expression of which decreased by 7–15 times. The E2F family of transcription factors play a key role in cell cycle control. As transcription activators, they interact with the promoters of the genes whose products are engaged in cell cycle regulation or DNA replication, and also control cell cycle progression. Along with the downregulation of the expression of E2F family transcription factors, the expression of other key cell cycle regulators was also significantly downregulated; the expression of many of them is under the control of E2F. These include cell division cycle genes (CDC6; CDC7; CDC20; CDC25A; CDC25B; CDC25C, and CDC45), which regulate the cell cycle at its different stages; cyclins, including A2, which controls both the G1/S and the G2/M transition phases of the cell cycle, and cyclin B1 (CCNB1) and cyclin B2 (CCNB2), which are indispensable in the control of the cell cycle at the G2/M (mitosis) transition; kinases, the regulators of the cell cycle, including cyclin-dependent kinase 1 (CDK1), checkpoint kinase 1 (CHEK1), polo-like kinase 1 (PLK1), and others; minichromosome maintenance complex components (MCM2-7); origin recognition complex subunits 1 and 6 (ORC1 and PRC6); and others. Thus, XMU-MP-1 triggers a cascade of transcription repression of the key cell cycle regulators in the Namalwa cells.

**Figure 4:**
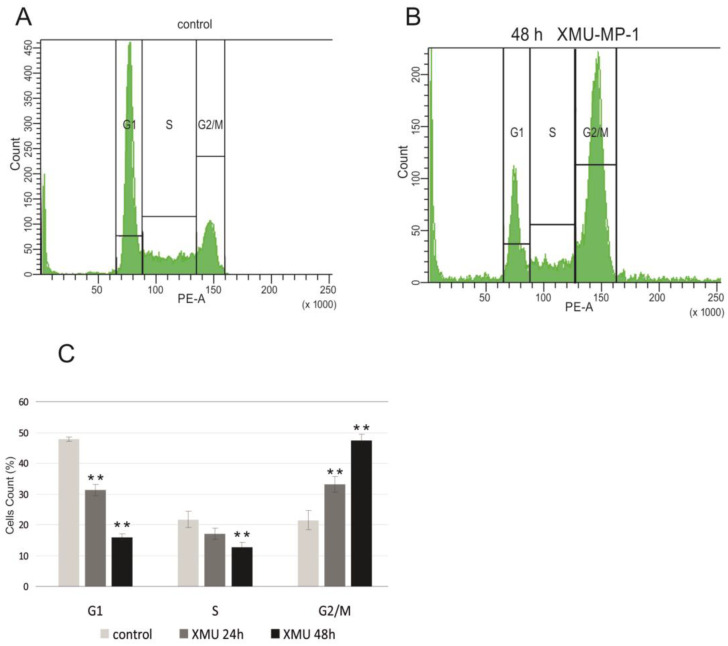
XMU-MP-1 contributes to cell cycle arrest in G2/M. Flow cytometry analysis in Namalwa cells. (**A**). Cells distribution across the cell cycle phases obtained by PI staining of non-treated cells. (**B**). Cells distribution across the cell cycle phases obtained by PI staining following the treatment with 0.6 µM XMU-MP-1. (**C**). Cell cycle analysis for the Namalwa cells, showing the mean percentage of cells in the G1, S, and G2/M phases. Bar heights reflect the mean percentages. Plots show the mean ± SEM for three independent biological experiments. *t*-tests were performed, and asterisks indicate a significant difference relative to the control cells (** *p* < 0.01).

To confirm the functional significance of these changes in the transcriptome, we performed cell cycle analysis using flow cytometry and showed that XMU-MP-1 significantly alters the cell cycle and leads to cell cycle arrest in the G2/M phase in Namalwa cells (Figure 4).

The results obtained from the RNA-seq and flow cytometry assays are in perfect agreement with the experiments that demonstrate the suppression of B- and T-tumor cell growth (Figure 1).

**Figure 5:**
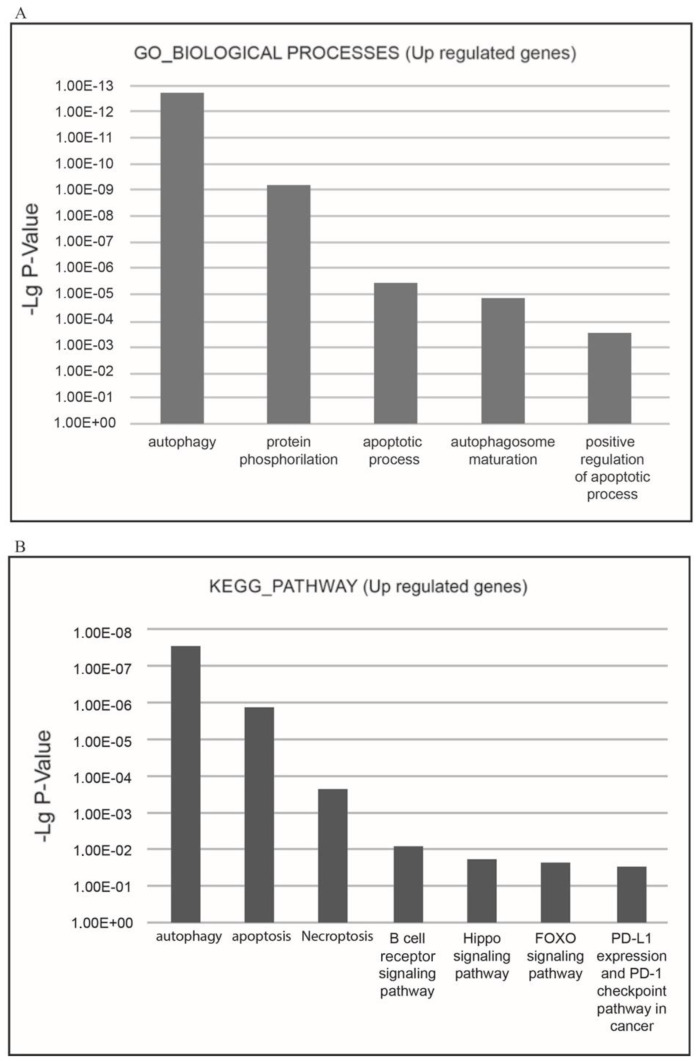
Functional enrichment analysis of upregulated differentially expressed genes (DEGs) in the XMU-MP-1-treated Namalwa cell line. (**A**) The most relevant BIOLOGICAL PROCESSES (Gene Ontology (GO) GOTERM BP FAT) and (**B**) the most relevant KEGG pathways.

The most relevant upregulated processes present in the KEGG pathways and GO_BIOLOGICAL PROCESSES databases are ‘autophagy’, ‘apoptosis’, and ‘necroptosis’, which confirms the suppressive effect of XMU-MP-1 on cell population growth, and demonstrates the mechanisms by which XMU-MP-1 regulates the programmed cell death (Figure 5A,B).

XMU-MP-1 induces changes in the level of mRNA in many key apoptosis regulatory genes (Supplementary Table S3). The ‘apoptosis’ term includes a significant group of upregulated DEGs involved in the development and regulation of apoptosis: CASP6 and CASP7 caspases; BBC3; BCL2A1; BLCAP; FAS (Fas cell surface death receptor); TRAF1; TRAF4; TNFSF10; TRADD; XAF1; APAF1; BIRC3; and others. The proteins encoded by these genes are essential for the development of apoptosis, including APAF1, a cytoplasmic protein that initiates apoptosis and forms the oligomeric complex, called an apoptosome, which activates caspases and triggers the caspase cascade; caspases 6 and 7; FAS, a cell surface death receptor that promotes apoptosis; pro-apoptotic proteins from the BCL2 family, such as BBC3 and BCL2A1; and apoptosis regulatory proteins, like MOAP1 and XAF1. MOAP1 interacts with BAX to activate caspase-dependent apoptosis, while XAF1 binds and inactivates apoptosis inhibitors (IAPs), leading to cell death.

**Figure 6:**
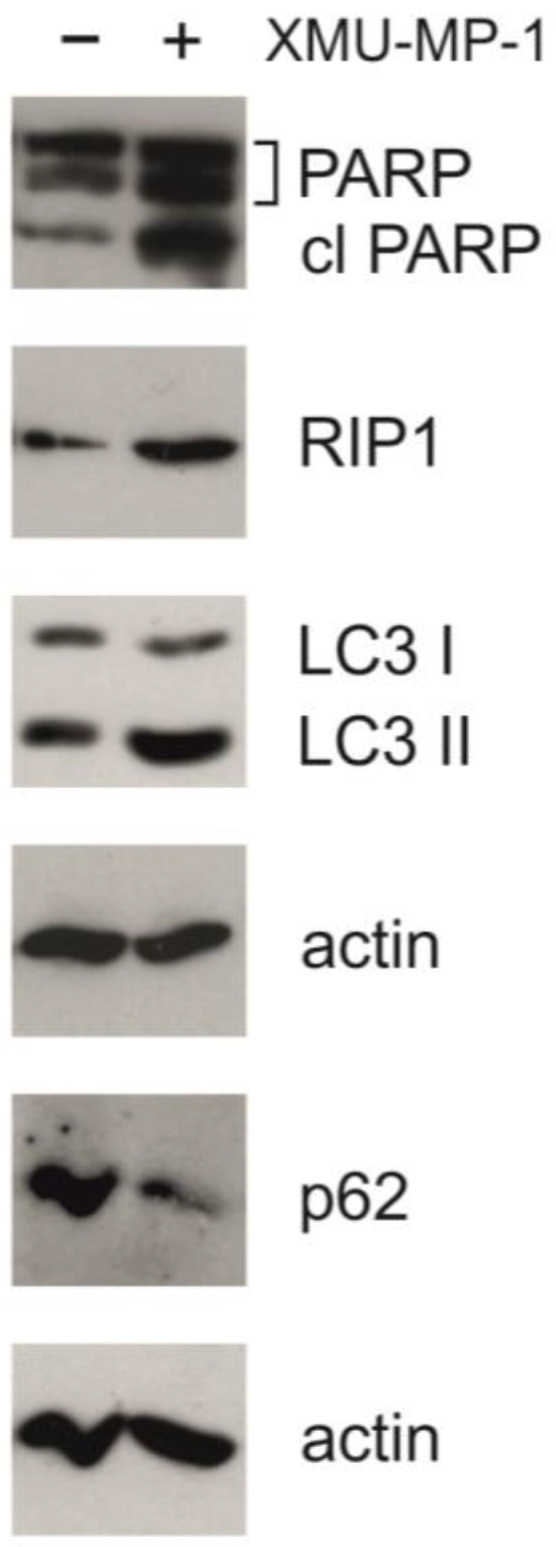
XMU-MP-1 activates programmed cell death. Western blot analysis in the untreated and XMU-MP-1-treated Namalwa cell line. Cells were incubated with 2.5 μM XMU-MP-1 for 24 h. In the control group, only DMSO was added to the medium. A total of 10 µg of protein extract was loaded into each well, beta-actin was used as a loading control.

The activation of apoptosis is further supported by the results of Western blot analysis. In response to the XMU-MP-1 treatment, an accumulation of the cleaved form of PARP-1 (poly(ADP-ribose)polymerase 1) was observed (Figure 6). This molecule is the downstream target of caspase-3 and can serve as an indicator of the activation of the caspase cascade and apoptosis [22]. This finding has also been confirmed by the results of the Caspase 3/7-Glo assay (Figure 2).

XMU-MP-1 considerably increases the expression of the key genes participating in autophagy activation and autophagosomes maturation (Supplementary Table S4). The ‘autophagy’ term includes a significant group of upregulated DEGs involved in autophagy regulation and autophagosome membrane formation, including beclin 1 (BECN1); DEPP autophagy regulator 1 (DEPP1); DNA damage-regulated autophagy modulator 2 (DRAM2); autophagy-related 2A (ATG2A); autophagy-related 4B cysteine peptidase (ATG4B); death-associated protein (DAP); VPS18 core subunit of CORVET and HOPS complexes (VPS18); VPS39 subunit of HOPS complex (VPS39); autophagy and beclin 1 regulator 1 (AMBRA1); ectopic P-granules 5 autophagy tethering factor (EPG5); and unc-51-like autophagy-activating kinase 1 (ULK1).

The activation of autophagy in the cells was confirmed by Western blot analysis. Figure 6 shows that under the XMU-MP-1 treatment, the p62/SQSTM1 protein (sequestosome 1) is degraded and the accumulation of LC3-II (microtubule-associated protein 1A/1B light-chain 3A) takes place. These two proteins are the markers of autophagy [22]. Additionally, we could observe an accumulation of the RIP1 protein, which may be linked to apoptosis, necroptosis, or autophagy [22]. These findings are consistent with the RNA-seq data showing changes in the transcriptome associated with programmed cell death and cell cycle defects.

Together, using the combination of Western blot, RNA-seq, and other assays, we have demonstrated that the decrease in the population numbers of hematopoietic tumor cells observed under the XMU-MP-1 treatment is caused by cytostatic and cytotoxic mechanisms.

We hypothesize that this cascade of changes begins with the activation of the Hippo signaling pathway and leads to the death of Namalwa cells. Under the effect of XMU-MP-1, the expression of some key genes in the Hippo signaling pathway changes. This change in expression suggests that the Hippo signaling pathway may be regulated through a feedback mechanism. XMU-MP-1 leads to an increase in the expression of several genes, including MST1 serine/threonine kinase 4, LIMD1 (LIM domain containing 1), MOB1A, MOB1B, NF2, and RASSF2.

## 3. Discussion

MST1/2 regulates the development of T and B cells through the “noncanonical” Hippo signaling pathway in hematopoiesis and hematopoietic tumor cells [23,24]. When the MST1 and MST2 kinase activities are inhibited, the canonical Hippo pathway leads to increased cell proliferation and decreased apoptosis in many somatic cells. However, the opposite effect has been observed in hematopoietic tumor cells [19].

We investigated how XMU-MP-1, the inhibitor of the serine/threonine kinases MST1/2, the key components of the Hippo pathway, affects hematopoietic tumors and demonstrated that the treatment of several hematopoietic tumor cell lines with XMU-MP-1 causes suppression of their growth and induces programmed cell death. This treatment causes significant changes in the transcriptome of these cells, while the expression of the “canonical” Hippo pathway target genes, such as Cyr61 (CCN1) and CTGF (CCN2), remains unchanged in the Namalwa cells under MST1/2 inhibition. These findings suggest that the Hippo pathway activates and regulates some alternative transcriptional pathways in hematological cancer cells

The results of whole-transcriptome analysis revealed that in the Namalwa cells, XMU-MP-1 suppresses the expression of genes whose products play a crucial role in regulating the cell cycle and DNA replication. It is known that imbalanced levels of proteins which regulate the cell cycle can lead to significant disruptions of mitosis and stop the cell cycle in the G2/M phase, which can trigger a mitotic catastrophe and subsequent cell death through apoptosis, necroptosis, or autophagy [25]. We observed similar effects in the Namalwa cells after XMU-MP-1 treatment. XMU-MP-1 induces cell cycle arrest at the G2/M phase and activates genes that regulate the three types of programmed cell death: apoptosis, autophagy, and necroptosis (Supplementary Tables S2–S5). This activation suggests that XMU-MP-1 has a specific effect on the death of hematopoietic tumor cells, but it does not affect triple-negative or triple-positive breast cancer cells, and does not cause their death.

The activation of various types of programmed cell death by XMU-MP-1 holds potential for treatment of hematological cancers, because the inhibition of programmed cell death is a strategy widely used by tumor cells to resist damaging factors. For example, the ability of neoplastic cells to escape apoptosis significantly increases their viability, making the activation of apoptosis essential for effective antitumor treatment.

The issue of autophagy appears to be more complex. It is known that autophagy plays a crucial role in maintaining the balance between proliferation and cell death of B and T lymphocytes. However, the cytotoxic role of autophagy has also been shown in cancer. There is abundant evidence that autophagy acts as a suppressor of neoplasm development [26,27].

Some effective chemotherapy drugs aim to activate one or more types of programmed cell death. For example, rasfonin triggers apoptosis, autophagy, and necroptosis in kidney cancer cells [28]. The antitumor activity of arsenic trioxide (As_2_O_3_) in acute promyelocytic leukemia depends on the activation of autophagy, leading to the death of the tumor cells [28,29]. RAD001 (everolimus), an mTOR inhibitor, also blocks cell survival in childhood acute lymphoblastic leukemia by inducing autophagy [30,31]. Resveratrol, another drug, induces the death of chronic myeloid leukemia cells through autophagy [32]. All these studies emphasize the significant role of autophagy in hematologic cancers. Our study has demonstrated that XMU-MP-1 activates the autophagy pathway by inducing the expression of genes involved in autophagosome assembly, including Beclin 1, DEP1, DAP, VPS18, and VPS39, and also upregulates the expression of AMBRA1.

The activation of necroptosis is also an important therapeutic tool for targeting tumors that are especially resistant to apoptosis. For example, the anticancer drug shikonin exerts an antitumor effect on osteosarcoma by triggering RIPK1- and RIPK3-dependent necroptosis [33]. Resibufogenin triggers necroptosis in colon cancer cells, thereby inhibiting tumor growth [34]. In our work, we have shown that expression of the RIPK1 protein significantly increased, and necroptosis regulator genes FAS, TNFSF10, TRADD, and RIPK1, as well as the expression of PEA15, the caspase 8 inhibitor, was activated in the presence of XMU-MP-1 in the Namalwa cell population (Supplementary Table S5).

We assume that treatment with XMU-MP-1 may increase the effectiveness of chemotherapy with a drug that damages DNA. In hematological tumors, it is known that DNA damage leads to the activation of a pro-apoptotic pathway through the nuclear relocation of ABL1 kinase. Previous studies have shown that low levels of YAP1 can block ABL1-induced apoptosis in hematological malignancies, while the genetic inactivation of MST1 restores YAP1 levels and causes cell death, both in vitro and in vivo. As a result, the simultaneous use of these two compounds may lead to an increase in the death rate of Namalwa cells.

It is possible that the combined use of chemotherapy drugs that damage DNA and XMU-MP-1 could reduce the effective dose of chemotherapy used in the treatment of hematological tumors and significantly reduce life-threatening toxic effects, such as free radical formation, which underlies cardiotoxicity, thrombocytopenia, leukopenia, and necrotizing colitis. XMU-MP-1 is known to stimulate platelet recovery after immune thrombocytopenia and promote the migration of megakaryocytes from the bone marrow [35]. It also promotes the regeneration of hematopoietic stem cells and progenitor cells and reduces the damage caused to the small intestine by whole-body radiation exposure, increasing the mean survival time of mice exposed to lethal radiation doses [20]. Our previous studies have also shown that XMU-MP-1 enhances the antitumor activity of other chemotherapy drugs, like etoposide and cisplatin, in hematologic tumor cells [21].

Therefore, XMU-MP-1 has the potential to be an effective component of combination therapy for hematologic tumors; however, further investigations are required.

## 4. Materials and Methods

### 4.1. Cell Lines

The following cell lines were used in the work: Namalwa, Daudi, and Ramos B lymphoblastoid Burkitt lymphomas, Jurkat T-cell lymphoblastoma, HL-60 acute promyelocytic leukemia, promyeloblast, K562 chronic myeloid leukemia at blast crisis, and MDA MB-231 and MCF-7 breast adenocarcinomas. Human cell lines were obtained from the Russian Cell Culture Collection, Institute of Cytology, St. Petersburg, Russia. Cells were maintained in DMEM or RPMI (GIBCO, Thermo Fisher Scientific, Waltham, MA, USA) with 10% FCS (FBS; HyClone, Logan, UT, USA), 100 U/mL penicillin, and 100 μg/mL streptomycin, in a 5% CO_2_ atmosphere.

### 4.2. Cell Proliferation Assay

Cells were cultured at +37 °C in a humid atmosphere in cultural plates manufactured by TRR (Zug, Switzerland) in DMEM or RPMI (Life Technologies, Carlsbad, CA, USA), with the addition of 10% calf fetal serum (BioSera, Cholet, France), in 5% CO_2_. The effect of XMU-MP-1 on Namalwa cell proliferation was evaluated using the CellTiter 96 Aqueous One Solution kit (Promega, USA). Namalwa cells were inoculated into a 96-well plate with a density of 3 × 10^4^ cells per well in the DMEM medium supplemented with 10% fetal calf serum 24 h prior to the addition of the studied compound. Antiproliferative activity of XMU-MP-1 was studied by treating Namalwa cells with the XMU-MP-1 reagent at the concentrations of 0.3; 0.6; 1.25; and 2.5 μM in DMSO. Cells with only DMSO and no XMU-MP-1 added served as the control. After 72 h of incubation, the antiproliferative and cytotoxic effects of XMU-MP-1 were analyzed using the CellTiter 96 Aqueous One Solution kit (Promega) according to the manufacturer’s protocol. Solutions’ optical density was measured at 490 nm using a Chameleon V plate reader (Hydex Oy, Finland). The amount of the formazan product calculated based on the absorbance rate at 490 nm is in direct proportion to the number of live cells in the culture. Cell viability was assessed relative to the control, and the concentration reducing cell viability by 50% (CD50) was determined.

### 4.3. Bioluminescence Caspase 3/7 Assay

Apoptosis caused by XMU-MP-1 in the cell lines was assessed using the Caspase 3/7-Glo kit (Promega). Namalwa cells were inoculated into 96-well plates with a density of 2 × 10^4^ cells per well in the DMEM or RPMI medium supplemented with 10% fetal calf serum 24 h prior to the addition of the studied compound. The apoptotic activity of XMU-MP-1 was studied by treating Namalwa cells with the XMU-MP-1 reagent at the concentrations of 0.3; 0.6; 1.25; and 2.5 μM. The other cell lines were treated with XMU-MP-1 at the concentration of 2.5 μM. Cells with only DMSO and no XMU-MP-1 added served as the control. After 48 h of incubation, apoptosis was assessed by measuring the activity of caspases 3 and 7 in accordance with the manufacturer’s protocol. Towards this end, 20 µL of reagent was added to each well, and cells were incubated for 30 min at room temperature. The luminescence of the solutions was then measured using a Chameleon V plate reader (Hydex Oy). Luminescence activity was directly proportional to the activity of the terminal capases 3 and 7 and to the cell apoptosis levels in the culture. The apoptosis levels at different concentrations of XMU-MP-1 was estimated based on the luminescence levels relative to the control.

### 4.4. Determination of Cell Cycle by Flow Cytometry

Cells were inoculated into 60 mm^2^ dishes and treated with 0.6 µM XMU-MP-1. Only DMSO was added to the control group. The measurements were taken in three separate sessions. Control cells were incubated for 48 h. XMU-MP-1-treated cells were incubated for 48 h. Cells treated with XMU-MP-1 for 24 h were first incubated for 24 h in the absence of XMU-MP-1, and then for an additional 24 h in its presence. Then, 24 h and 48 h after the treatment, cells were collected, fixed in 70% ethanol, and stained with 50 µg/mL propidium iodide (PI) treated with 100 µg/mL RNase A (Sigma, catalog R-4875) at 37 °C for 60 min. Flow cytometric analysis was carried out using the BD LSRFortessa cell analyzer equipped with the BD Bioscience software v9.0 (Becton Dickinson, San Jose, CA, USA). The distribution of cells according to their fluorescence was determined for G1, S, and G2/M subpopulations.

### 4.5. Antibodies and Western Blot Analysis

The following antibodies were used: rabbit anti-PARP (Cell Signaling, Danvers, MA, CST 9541) and rabbit anti-cleaved PARP (CST 137653), rabbit anti-beta-actin (CST 13E5), rabbit anti-LC3 (CST 12741), and rabbit anti-p62 (CST B0255). Additionally, rabbit anti-RIP1 (CST 94C12) and goat anti-Rabbit HRP (Jackson ImmunoResearch, 111-035-144) antibodies were utilized. Protein extracts (10 µg) were mixed with the loading buffer containing DTT and incubated at 40 °C for 10 min. The samples were then applied onto the 8% or 12% SDS-PAGE and transferred to nitrocellulose membranes after separation (GE Healthcare). Membranes were blocked with 5% nonfat milk in TBS for 1 h at room temperature. A total of 10 µg of cell protein extract was loaded into each lane, and beta-actin was used as the loading control. The membranes were probed with the primary antibodies overnight at 4 °C and then washed three times for 15 min with TBS-T. After that, they were incubated for 1 h at RT with anti-rabbit HRP antibodies (Santa Cruz Biotechnology, sc-2005) at a 1:5000 dilution. After four additional washing steps with TBS-T, signal detection was performed using the ECL reagent according to the standard protocol (GE Healthcare). At least two independent Western blot analyses were conducted for each protein.

### 4.6. Sequencing Library Preparation

The samples of the human Namalwa cells treated with 0.3 or 2.5 µM of XMU-MP-1 for 72 h and the control Namalwa cells to which only DMSO was added were obtained in triplicate. The NEBNext Poly(A) mRNA Magnetic Isolation Module (NEB#E7490) was used to isolate mRNA. NEBNext Ultra II Directional RNA Library Prep Kit for Illumina (NEB #E7760) was used with 3 ug of total RNA for sequencing library construction. Standard Illumina protocols were used to make RNA libraries. AgencourtAMPure XP beads were used to purify double-stranded cDNA, ligation reaction products, and PCR reaction products.

### 4.7. NGS Sequencing and Data Processing

The KAPA Library Quant qPCR kit (Kapa Biosystems) was used to measure molar library concentrations. Library size distribution was assessed after PCR (12 cycles) using Agilent BioAnalyzer (Santa Clara, CA, USA). Then, the cDNA libraries were normalized and pooled together in equal volumes. Finally, they were sequenced using 150 base-pair double-ended reads with the Illumina NovaSeq instrument (Illumina, San Diego, CA, USA). The bcl2fastq v2.20 Conversion Software (Illumina) was utilized to obtain FASTQ files. The hisat software v2.2.1 was used to map reads on the human genome (hg38). In each library, about 89–90% of the obtained data on average were uniquely aligned. The htseq-count package was used to calculate the number of reads which were mapped to known genes (ncbi—entrezID). The obtained values (cpm—countpermillion) for each gene in each library were combined into a matrix for further analysis. Filtration, normalization by the TMM method, variance estimation, and assessment of differentially expressed genes were performed using the edgeR module. A quasi-likelihood F-test (default in edgeR) was used to assess the statistical significance of the observed expression changes. The Benjamini–Hochberg method was then applied to the resulting *p*-values to calculate the false discovery rate (FDR).

### 4.8. Functional Enrichment Analysis of DEGs

Gene Ontology (GO) screening was performed using DAVID (https://davidbioinformatics.nih.gov/home.jsp (accessed on 28 February 2025)) including GOTERM_BP_FAT (biological process), GOTERM_ MF_FAT (molecular function), GOTERM_CC_FAT (cellular component), and KEGG pathway (https://www.genome.jp/kegg/pathway.html (accessed on 28 February 2025)) resources. DAVID calculates the modified Fisher’s exact *p*-values to demonstrate GO or molecular pathway enrichment. A Paji <0.01 was chosen as the cut-off criterion.

### 4.9. Statistics

Statistical analysis of the results from the cell proliferation assay, the Bioluminescence Caspase 3/7 assay, and the flow cytometry assay was conducted using the GraphPad software v10 (GraphPad Software, San Diego, CA, USA). Student’s *t*-tests were used to generate *p*-values. Error bars represent the standard error of the mean (S.E.M.). (* *p* < 0.05 and ** *p* < 0.01).

### 4.10. Accession Number

RNA-Seq data GSE279247.

## 5. Conclusions

Taking into account the results obtained in our work, we consider the investigation of the possibility of using the MST1/2 kinase inhibitors in the treatment of hematologic tumors extremely promising.

## Data Availability

Data is contained within the article and provided by GEO Database GSE80287.

## Supplementary Materials

The following supporting information can be downloaded at: https://www.mdpi.com/article/10.3390/ph18060874/s1, Figure S1. Relative level of the MST1(STK4) mRNA in human cell lines measured by Real-Time PCR. The graphs show means +/− S.E.M. of three independent experiments; Figure S2. Effect of XMU-MP-1 on Namalwa cell growth rate. Cells were inoculated into 6-well plates at 500,000 cells/well in the final volume of 2 mL complete DMEM in four replicates each line and each time point. The plots show mean ± S.E.M. for four independent experiments; Figure S3. Original blot images for all the assessed proteins. Namalwa cells; Table S1. EC50 is achieved in the Namalwa, Raji, Ramos, Jurkat, and Daudi cell lines in 72 h at the XMU-MP-1 concentrations ranging from 1.20 to 2.7 μM; Table S2. Cell Cycle DEGs under XMU-MP-1 treatment 2.5 μM and 0.3 μM; Table S3. Apoptosis DEGs under XMU-MP-1 treatment 2.5 μM and 0.3 μM; Table S4. Autophagy DEGs under XMU-MP-1 treatment 2.5 μM and 0.3 μM; Table S5. Necroptosis DEGs under XMU-MP-1 treatment 2.5 μM and 0.3 μM.
