# Supplementary material for: XMU-MP-1, Inhibitor of STE20-like MST1/2 Kinases of the Hippo Signaling Pathway, Suppresses the Cell Cycle, Activates Apoptosis and Autophagy, and Induces Death of Hematopoietic Tumor Cells
Source: Pharmaceuticals (Basel). 2025 Jun 12;18(6):874. doi: 10.3390/ph18060874 (PMC12196308; doi:10.3390/ph18060874)

Supplementary Materials

XMU-MP-1, Inhibitor of STE20-like MST1/2 Kinases of the Hippo Signaling Pathway, Suppresses the Cell Cycle, Activates Apoptosis and Autophagy, and Induces Death of Hematopoietic Tumor Cells

Alexander G. Stepchenko, Sofia G. Georgieva and Elizaveta V. Pankratova

Figure S1.

Relative level of the MST1(STK4) mRNA in human cell lines measured by Real-Time PCR. The graphs show means +/- S.E.M. of three independent experiments.

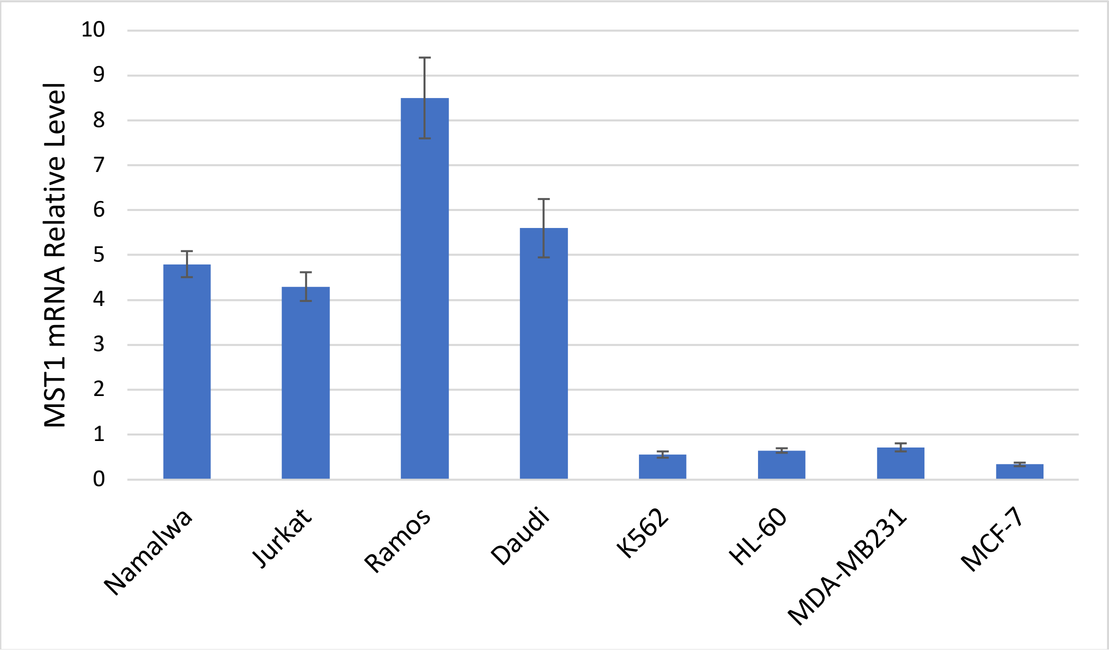

We compared the MST1 mRNA levels in hematopoietic cell lines (Namalwa, Jurkat, Daudi, Ramos, K562, HL-60) and in the breast cancer cell lines (MDA-MB231, MCF-7) by qRT-PCR using the corresponding primers. Our results show that there is a high level of MST1 expression in Namalwa, Daudi and Ramos B-cell lymphoblastomas and Jurkat T-cell lymphoblastoma.

RNA from cell lines was purified using Trizol. Reverse transcription was performed using the RevertAid H Minus First Strand cDNA Synthesis Kit (Thermo Scientific) and qPCR was performed using the qPCRmix-HS-SYBR kit (Evrogen, Russia). Primers used are as follows:
MST1-F; 5’-CGCCGGCAGCTGAAAAAGTT-3’;
MST1-R; 5’-GCCCCACAGTACTCCATAAC-3’;
mRNA levels were normalized to that of the GUS gene GUS-Forw 5’-cgtggttggagagctcatttgga-3’; GUS-Rev 5’-attccccagcactctcgtcggt-3’. Measurements at each point were made in at least three replicates, and the mean values were calculated.

Table S1. EC50 is achieved in the Namalwa, Raji, Ramos, Jurkat, and Daudi cell lines in 72 h at the XMU-MP-1 concentrations ranging from 1.20 to 2.7 µM

Cell line	EC_{50}	Mean +/-SD
Ramos	1.21 µM	1.21+/- 0.1
Namalwa	1.72 µM	1.72 +/- 0.094
Jurkat	1.85 µM	1.85 +/- 0.125
Daudi	2.59 µM	2.59 +/- 0.148

Figure S2. Effect of XMU-MP-1 on Namalwa cell growth rate. Cells were inoculated into 6-well plates at 500 000 cells/well in the final volume of 2 ml complete DMEM in four replicates each line and each time point. The plots show mean ± S.E.M. for four independent experiments.

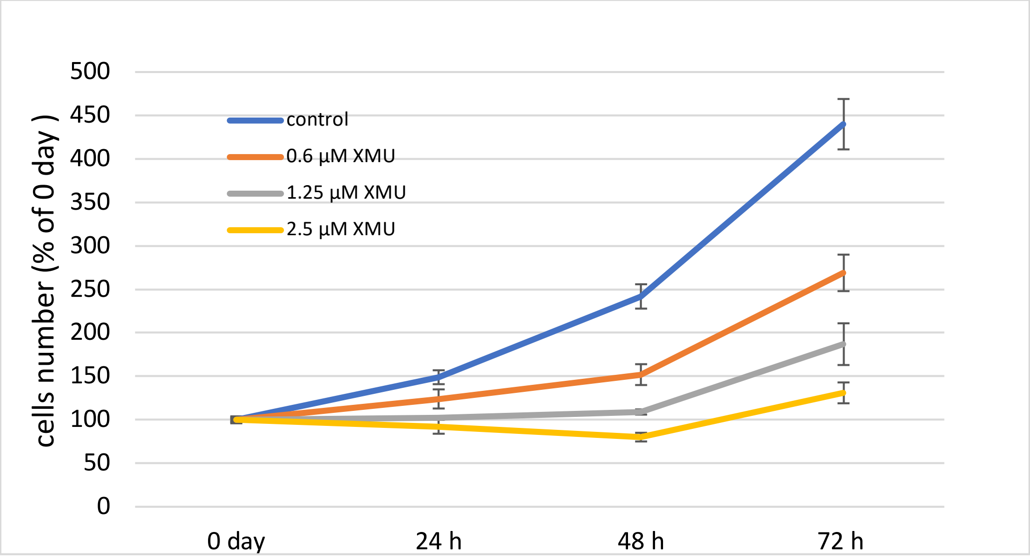

Supplementary Table S2. Cell Cycle DEGs under XMU-MP-1 treatment 2.5 µM and 0.3 µM

Fog2FoldChange: Base 2 logarithm of the normalized read count ratio change in the compared samples. Values greater than 0 indicate an increase in expression in the experimental group relative to the control group; values less than 0 indicate a decrease.
Paji – Benjamini-Hochberg multiple comparison corrected (FDR) value (a statistical value expressed as a number between 0 and 1 that is used to assess significance)

hgnc_symbol	Gene	DEGs XMU 2.5 µM log2FoldChange	paji	DEGs XMU 0.3 µM log2FoldChange	paji
BUB1B	BUB1 mitotic checkpoint serine/threonine kinase	-3,110550126	1,1994E-164	-1,181060904	7,7394E-32
DBF4	DBF4-CDC7 kinase regulatory subunit	-1,230177513	7,83775E-29	-0,684705432	7,1657E-10
DBF4B	DBF4B-CDC7 kinase regulatory subunit	-1,609978934	2,95568E-24	-0,59689319	0,0001658
BUB1	BUB1 mitotic checkpoint serine/threonine kinase	-2,17708425	2,4926E-103	-0,613092443	2,6193E-10
E2F1	E2F transcription factor 1	-2,148441425	4,95883E-88	-1,220028451	2,5063E-32
E2F2	E2F transcription factor 2	-3,951234517	1,25E-194	-1,969745673	1,251E-127
FBXO5	F-box protein 5	-1,290711669	9,53004E-19	-0,825042941	1,5658E-08
MTBP	MDM2 binding protein	-1,78037821	1,9741E-10	-0,766867433	0,0066246
MYC	MYC proto-oncogene, bHLH transcription	-1,790717142	1,8822E-194	-0,704124785	1,6153E-32
NDC80	NDC80 kinetochore complex component	-2,640150994	5,30052E-77	-1,058828078	8,5108E-17
PTTG1	PTTG1 regulator of sister chromatid separation, securin	-1,840451829	9,17813E-77	-0,83872911	5,7271E-18
RAD21	RAD21 cohesin complex component	-1,054485114	2,114E-42	-0,952621532	1,2824E-34
RBL1	RB transcriptional corepressor like 1	-1,824222834	4,68624E-17	-1,200573114	1,3222E-08
TICRR	TOPBP1 interacting checkpoint and replication regulator	-2,965230593	2,11403E-74	-1,235314438	8,1598E-18
TTK	TTK protein kinase	-2,542177778	4,27176E-57	-0,946510529	5,9891E-11
WEE1	WEE1 G2 checkpoint kinase	-2,310818427	4,901E-113	-1,333191851	3,6396E-45
AURKB	aurora kinase B	-3,342604797	6,0402E-142	-1,485948521	2,5218E-40
CDC20	cell division cycle 20	-2,814738356	7,6002E-205	-1,10482565	2,1676E-41
CDC25A	cell division cycle 25A	-2,381240657	8,23834E-52	-1,268568623	6,5446E-18
CDC25B	cell division cycle 25B	-1,858348436	1,7094E-107	-0,878604141	1,6718E-26
CDC25C	cell division cycle 25C	-2,832112383	1,92852E-28	-1,221640728	1,2915E-07
CDC6	cell division cycle 6	-2,258493667	6,66119E-76	-1,260664869	1,8213E-27
CDC7	cell division cycle 7	-1,808775206	1,25531E-23	-0,956235211	7,7515E-08
CDC45	cell division cycle 45	-2,781385406	2,24504E-61	-1,325274011	6,1349E-18
CDCA5	cell division cycle associated 5	-2,933482125	3,2351E-164	-1,516968574	4,2565E-55
CHEK1	checkpoint kinase 1	-2,756495279	5,11894E-79	-1,397717099	5,8785E-27
CDT1	chromatin licensing and DNA replication factor 1	-3,887241152	1,5506E-293	-2,541428548	1,65E-159
CCNA2	cyclin A2	-3,040641353	1,4308E-195	-1,256985874	3,2258E-43
CCNB1	cyclin B1	-2,335533921	6,9298E-105	-0,663259648	6,7924E-11
CCNB2	cyclin B2	-2,43724124	2,1706E-116	-0,763708512	8,8344E-15
CDK1	cyclin dependent kinase 1	-2,88163362	1,22412E-84	-1,580127758	2,5027E-32
CDKN1C	cyclin dependent kinase inhibitor 1C	-3,904226117	1,42342E-25	-3,494338037	5,7985E-23
ESCO2	establishment of sister chromatid cohesion N-acetyltransferase 2	-1,820164746	7,22147E-10	-0,765006511	0,0093457
ESPL1	extra spindle pole bodies like 1, separase	-3,562881852	2,43E-271	-1,477566285	3,9749E-84
MCM2	minichromosome maintenance complex component 2	-2,016786707	8,2147E-191	-1,051447792	4,3574E-57

MCM3	minichromosome maintenance complex component 3	-1,421878741	1,46108E-97	-0,837112319	1,5896E-35
MCM4	minichromosome maintenance complex component 4	-2,512872968	5,0941E-299	-1,394521952	6,223E-104
MCM5	minichromosome maintenance complex component 5	-2,250779437	1,1279E-247	-1,384813603	2,669E-102
MCM6	minichromosome maintenance complex component 6	-1,67834259	1,1613E-120	-0,895130716	1,4628E-37
MCM7	minichromosome maintenance complex component 7	-2,122416093	3,0874E-274	-1,326349749	3,265E-112
MAD1L1	mitotic arrest deficient 1 like 1	-1,94260009	4,29132E-22	-1,589635736	7,9145E-16
MAD2L1	mitotic arrest deficient 2 like 1	-2,429340344	2,64055E-59	-1,159655989	7,7001E-17
ORC1	origin recognition complex subunit 1	-2,993036811	9,46581E-83	-1,568574305	1,8344E-30
ORC6	origin recognition complex subunit 6	-1,982417537	2,06551E-28	-1,323000395	2,4952E-14
PLK1	polo like kinase 1	-3,046550009	4,6587E-281	-1,314083051	1,0962E-66
PCNA	proliferating cell nuclear antigen	-1,354280112	4,2747E-78	-0,705555302	1,5872E-22
PKMYT1	protein kinase, membrane associated tyrosine/threonine 1	-1,839918553	6,59836E-77	-1,126439198	6,1435E-32
SGO1	shugoshin 1	-2,728277174	5,14928E-22	-1,042155932	3,7575E-05
TRIP13	thyroid hormone receptor interactor 13	-2,393598035	1,08115E-37	-1,121576936	7,1193E-11

Supplementary Table S3. Apoptosis DEGs under XMU-MP-1 treatment 2.5 µM and 0.3 µM

Fog2FoldChange: Base 2 logarithm of the normalized read count ratio change in the compared samples. Values greater than 0 indicate an increase in expression in the experimental group relative to the control group; values less than 0 indicate a decrease.
Paji – Benjamini-Hochberg multiple comparison corrected (FDR) value (a statistical value expressed as a number between 0 and 1 that is used to assess significance)

**hgnc_ symbol**		**XMU 2.5 µM log2FoldChange**	**padj**	**XMU 0.3 µM log2FoldChange**	**padj**
BBC3	BCL2 binding component 3	1,519026091	2,2291E-26	0,5372746	0,001026306
BCL2A1	BCL2 related protein A1	2,021224222	2,7877E-58	1,5243966	6,28387E-32
FAS	Fas cell surface death receptor	1,487112577	2,0473E-12	0,9218671	6,14949E-05
MCL1	MCL1 apoptosis regulator, BCL2 family	1,233741067	2,145E-113	1,1669036	5,4307E-101
TNFSF10	TNF superfamily member 10	1,908842426	1,9078E-10	1,5865399	3,65736E-07
TRADD	TNFRSF1A associated via death domain	1,524605569	4,9055E-20	1,0452943	1,96831E-09
APAF1	apoptotic peptidase activating factor 1	2,177845308	1,81E-64	1,7790445	4,60665E-42
CASP6	caspase 6	1,321189724	1,7031E-09	0,7075422	0,003611924
CASP9	caspase 9	0,763389116	1,1637E-09	0,5604583	1,96162E-05
CASP3	caspase 3	0,642425197	1,8766E-06	0,5646789	4,74702E-05
CASP7	caspase 7	1,298805699	6,358E-14	1,1717345	3,43976E-11
GADD45A	growth arrest and DNA damage inducible alpha	2,936910652	1,2E-241	2,1336276	6,9564E-181
GADD45B	growth arrest and DNA damage inducible beta	1,261337016	1,039E-41	0,6673573	1,05931E-11
HRK	harakiri, BCL2 interacting protein	1,631597114	8,0735E-80	1,3720557	2,28239E-55
RIPK1	receptor interacting serine/threonine kinase 1	1,03740238	4,1836E-09	0,9171099	4,13378E-07
TNF	tumor necrosis factor	1,969231966	1,1846E-31	1,7214525	1,20153E-23
MOAP1	modulator of apoptosis 1	1,450443299	9,8369E-12	1,0074362	8,11601E-06

Supplementary Table S4. Autophagy DEGs under XMU-MP-1 treatment 2.5 µM and 0.3 µM

Fog2FoldChange: Base 2 logarithm of the normalized read count ratio change in the compared samples. Values greater than 0 indicate an increase in expression in the experimental group relative to the control group; values less than 0 indicate a decrease.
Paji – Benjamini-Hochberg multiple comparison corrected (FDR) value (a statistical value expressed as a number between 0 and 1 that is used to assess significance)

Hgnc symbol	gene	DEGs X XMU 2.5µM log2FoldChange	padj	DEGs XMU 0.3µM log2FoldChange	padj
DEPP1	DEPP autophagy regulator 1	5,338854339	1,4662E-287	4,313814853	4,2684E-184
TECPR1	tectonin beta-propeller repeat containing 1	0,94625953	3,40865E-33	0,652976168	9,24644E-16
TECPR2	tectonin beta-propeller repeat containing 2	1,418645743	4,65935E-50	1,077854681	2,27587E-28
TP53INP1	tumor protein p53 inducible nuclear protein 1	1,30634982	1,00735E-33	1,15736948	3,15082E-26
C9orf72	C9orf72-SMCR8 complex subunit	0,610707515	0,000794713	0,392531237	0,051262984
GABARAPL1	GABA type A receptor associated protein like 1	1,557790938	1,4598E-58	1,146174794	7,47947E-31
GABARAPL2	GABA type A receptor associated protein like 2	0,472605002	1,48224E-07	0,27386405	0,004935548
NBR1	NBR1 autophagy cargo receptor	0,440748557	1,1751E-07	0,350787684	4,85397E-05
RB1CC1	RB1 inducible coiled-coil 1	0,427913121	0,000903648	0,043165835	0,833640931
SH3GLB1	SH3 domain containing GRB2 like, endophilin B1	0,370108102	0,001024195	0,266127163	0,027645728
SUPT20H	SPT20 homolog, SAGA complex component	0,622804961	1,46741E-09	0,634397825	9,97305E-10
TAX1BP1	Tax1 binding protein 1	0,470659206	1,30735E-09	0,263173292	0,001614728
VPS16	VPS16 core subunit of CORVET and HOPS complexes	0,529041865	5,53379E-07	0,487118058	6,443E-06
VPS18	VPS18 core subunit of CORVET and HOPS complexes	0,524488308	6,1343E-09	0,337692602	0,000434767
VPS33A	VPS33A core subunit of CORVET and HOPS complexes	0,398846734	0,003295222	0,371216028	0,007806806
VPS39	VPS39 subunit of HOPS complex	0,998110982	3,8397E-53	0,777705184	7,01034E-32
VPS41	VPS41 subunit of HOPS complex	0,359870263	0,000199148	0,277272763	0,006519813
WDR45	WD repeat domain 45	0,701161111	0,004084301	0,376271047	0,185804386
AMBRA1	utophagy and beclin 1 regulator 1	0,596654664	1,76879E-12	0,45251675	2,29299E-07
ATG13	autophagy related 13	0,259633343	0,001789599	0,06237397	0,584784332
ATG14	autophagy related 14	0,726306314	0,000286175	0,484465316	0,026597551
ATG16L2	autophagy related 16 like 2	0,538343465	4,52486E-06	0,304048991	0,019023461

ATG2A	autophagy related 2A	0,712132482	2,17964E-15	0,570948359	5,72497E-10
ATG4B	autophagy related 4B cysteine peptidase	0,621748516	1,91339E-14	0,452806489	7,27983E-08
BECN1	beclin 1	0,838589519	3,04179E-21	0,588680714	1,45988E-10
DAPK3	death associated protein kinase 3	0,621941623	4,14019E-12	0,351380319	0,000260449
EPG5	ectopic P-granules 5 autophagy tethering factor	0,753414739	3,3182E-15	0,52789637	1,13571E-07
LAMP1	lysosomal associated membrane protein 1	0,334640362	0,001755533	0,15028044	0,23941671
MAP1LC3B	microtubule associated protein 1 light chain 3 beta	0,240504511	0,002057179	0,005185892	0,969407222
NRBF2	nuclear receptor binding factor 2	0,39461986	0,000335484	0,404993248	0,000274061
OPTN	optineurin	0,979926629	7,47558E-20	0,817024826	1,08637E-13
PIK3C3	phosphatidylinositol 3-kinase catalytic subunit type 3	0,446467515	0,000844589	0,343375235	0,015593624
PLEKHM1	pleckstrin homology and RUN domain containing M1	0,895887418	4,83332E-20	0,582813569	9,9995E-09
STX17	syntaxin 17	0,967020281	2,56144E-10	0,936422453	1,53328E-09
STX7	syntaxin 7	0,395012288	8,50727E-06	0,267026493	0,004761995
TP53INP2	tumor protein p53 inducible nuclear protein 2	0,460799242	1,79024E-07	0,244782903	0,012012239
ULK1	unc-51 like autophagy activating kinase 1	1,527681415	3,35283E-41	1,249157657	4,0775E-27
TFEB	transcription factor EB	0,431654234	7,04362E-10	0,117312587	0,174081719
TMEM59	transmembrane protein 59	0,588727462	2,33059E-10	0,454143929	2,38963E-06
TRIM5	tripartite motif containing 5	1,158084741	9,39793E-25	1,034921057	1,70924E-19
TP53INP1	tumor protein p53 inducible nuclear protein 1(	1,306349823	1,00735E-33	1,157369481	3,15082E-26
UBQLN2	ubiquilin 2	0,400630334	0,000196876	0,42245281	9,77676E-05
VCP	valosin containing protein	1,115726317	2,11215E-90	0,963028608	7,038E-67

Supplementary Table S5. Necroptosis DEGs under XMU-MP-1 treatment 2.5 µM and 0.3 µM

Fog2FoldChange: Base 2 logarithm of the normalized read count ratio change in the compared samples. Values greater than 0 indicate an increase in expression in the experimental group relative to the control group; values less than 0 indicate a decrease.
Paji – Benjamini-Hochberg multiple comparison corrected (FDR) value (a statistical value expressed as a number between 0 and 1 that is used to assess significance)

Hgnc symbol	gene	DEGs X XMU 2.5µM log2FoldChange	Padj	DEGs X XMU 0.3µM log2FoldChange	padj
JAK3	Janus kinase 3	1,22580971	7,31857E-63	1,08531412	8,58376E-49
TNFSF10	TNF superfamily member 10	1,908842426	1,90785E-10	1,586539904	3,65736E-07
TRADD	TNFRSF1A associated via death domain	1,524605569	4,9055E-20	1,045294252	1,96831E-09
BIRC3	baculoviral IAP repeat containing 3	1,892512957	1,3499E-126	1,500302353	1,97971E-78
CHMP7	charged multivesicular body protein 7	1,386964694	2,3258E-105	1,264129019	1,07634E-86
CYBB	cytochrome b-245 beta chain	1,008007054	3,84324E-25	1,246509535	3,28833E-38
PYGB	glycogen phosphorylase B	1,018326641	5,73718E-33	0,750153605	1,00664E-17
IRF9	interferon regulatory factor 9	1,402353524	4,46436E-14	0,749930204	0,00023359
PLA2G4C	phospholipase A2 group IVC	2,547046884	3,5395E-24	1,807598789	6,92782E-12
RIPK1	receptor interacting serine/threonine kinase 1	1,03740238	4,18355E-09	0,917109853	4,13378E-07
STAT1	signal transducer and activator of transcription 1	1,658961532	2,472E-136	1,262213181	2,86821E-77
SMPD1	sphingomyelin phosphodiesterase 1	1,110645129	1,62216E-11	0,805059432	3,25674E-06
TNF	tumor necrosis factor	1,969231966	1,18459E-31	1,721452451	1,20153E-23
CFLAR	CASP8 and FADD like apoptosis regulator	0,981705972	4,31145E-16	0,563222232	1,33046E-05
CYLD	CYLD Lysine 63 Deubiquitinase	0,659690848	1,56561E-14	0,537778796	1,04426E-09
DNM1L	dynamin 1 like	0,325819906	0,003976246	0,181903034	0,158438578
RIPK1	receptor interacting serine/threonine kinase 1	1,03740238	4,18355E-09	0,917109853	4,13378E-07
RNF31	ring finger protein 31	0,912683944	0,000234752	0,481741251	0,092552529

Figure S3. Original blot images for all the assessed proteins. Namalwa cells.

RIP1 and actin. Lines 1 and 3 – XMU-MP-1 2.5 µM 24 hours, 2 -control cells

1. 2. 3. 1. 2. 3

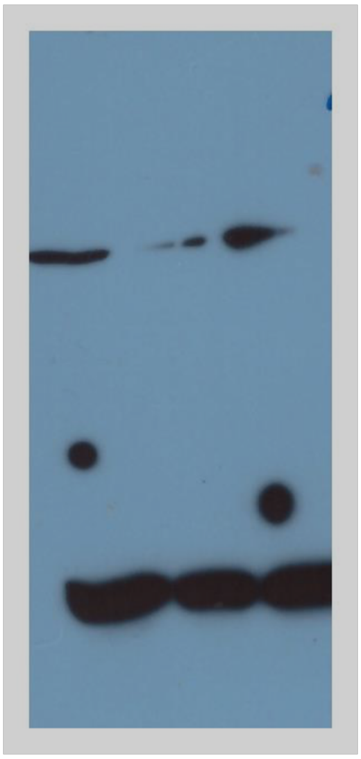

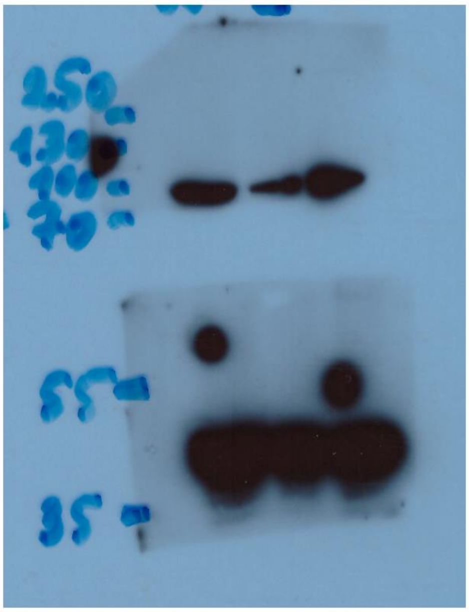

p62. Lines 1 and 3 – XMU-MP-1 2.5 µM 24 hours, 2 -control cells

1. 2. 3.

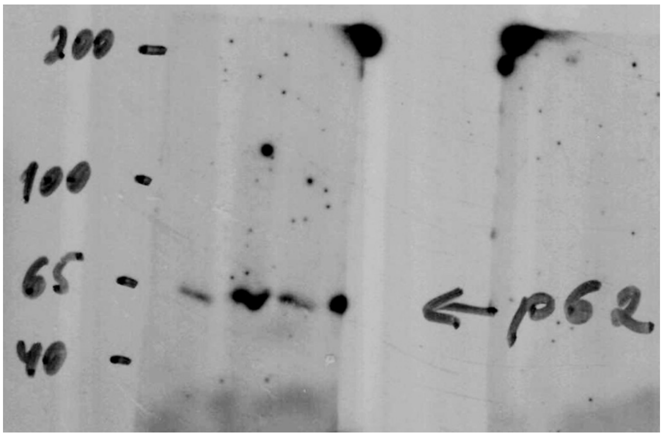

Actin (45 kDa)

1. 2. 3.

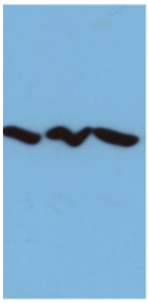

LC3 I and LC3 II . Lines 1 – XMU-MP-1 2.5 µM 24 hours, 2 -control cells

1. 2.

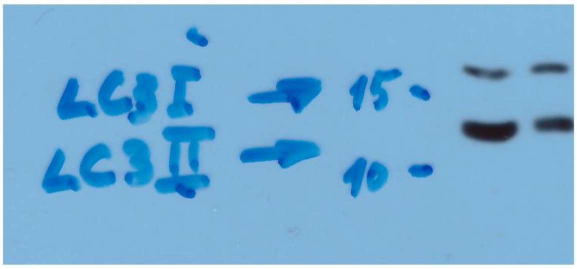

PARP and cleaved PARP. Lines 1 and 3 – XMU-MP-1 2.5 µM 24 hours, 2 -control cells. (clPARP – 90 kDa; PARP – 120-130 kDa)

1. 2. 3.

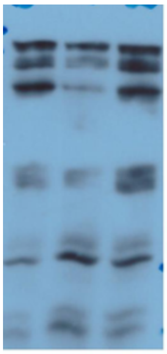

2. 3.

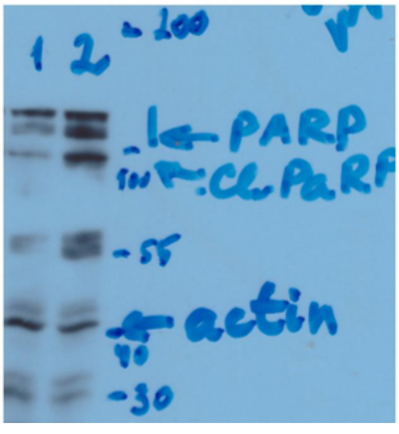

Supplement: Supplementary file 1 [file pharmaceuticals-18-00874-s001.zip › pharmaceuticals-3533433-supplementary.pdf]
